# High Yield Production Process for *Shigella* Outer Membrane Particles

**DOI:** 10.1371/journal.pone.0035616

**Published:** 2012-06-06

**Authors:** Francesco Berlanda Scorza, Anna Maria Colucci, Luana Maggiore, Silvia Sanzone, Omar Rossi, Ilaria Ferlenghi, Isabella Pesce, Mariaelena Caboni, Nathalie Norais, Vito Di Cioccio, Allan Saul, Christiane Gerke

**Affiliations:** 1 Novartis Vaccines Institute for Global Health, Siena, Italy; 2 Novartis Vaccines and Diagnostics, Siena, Italy; Charité-University Medicine Berlin, Germany

## Abstract

Gram-negative bacteria naturally shed particles that consist of outer membrane lipids, outer membrane proteins, and soluble periplasmic components. These particles have been proposed for use as vaccines but the yield has been problematic. We developed a high yielding production process of genetically derived outer membrane particles from the human pathogen *Shigella sonnei*. Yields of approximately 100 milligrams of membrane-associated proteins per liter of fermentation were obtained from cultures of *S. sonnei* Δ*tolR* Δ*galU* at optical densities of 30–45 in a 5 L fermenter. Proteomic analysis of the purified particles showed the preparation to primarily contain predicted outer membrane and periplasmic proteins. These were highly immunogenic in mice. The production of these outer membrane particles from high density cultivation of bacteria supports the feasibility of scaling up this approach as an affordable manufacturing process. Furthermore, we demonstrate the feasibility of using this process with other genetic manipulations e.g. abolition of O antigen synthesis and modification of the lipopolysaccharide structure in order to modify the immunogenicity or reactogenicity of the particles. This work provides the basis for a large scale manufacturing process of Generalized Modules of Membrane Antigens (GMMA) for production of vaccines from Gram-negative bacteria.

## Introduction

*Shigella* spp. are Gram-negative bacteria that infect the intestinal epithelium and cause dysentery. In 1999 the World Health Organization estimated an annual burden of 164.7 million shigellosis cases throughout the year of which 163.2 occur in developing countries, including 1.1 million deaths, mostly in children younger than 5 years of age [1]. Four serogroups have been identified: *S. dysenteriae* (15 serotypes), *S. boydii* (20 serotypes), *S. flexneri* (14 serotypes) and *S. sonnei* (1 serotype) [2]. No vaccine is currently available. So far, vaccine candidates based on O antigen conjugates and live attenuated strains have been shown in clinical trials to protect against homologous strains [2]–[6]. Vaccines using inactivated bacteria or subcellular components are at various stages of development [3], [6].

Gram-negative bacteria naturally shed outer membrane particles consisting of outer membrane lipids, outer membrane proteins, and enclosed periplasmic proteins [7]–[9]. Unlike most unilamellar biological vesicles, outer membrane particles are formed by blebbing and not by invagination of the membrane. Thus, the orientation of components in the membrane of the outer membrane particles is the same as in the bacterial outer membrane and the components in the outer face of the bacterial outer membrane are also in the outer face of the outer membrane particles [7]. Outer membrane particles are naturally shed at low concentration. Mutations such as the deletion of gene *gna33* in *Neisseria meningitidis* [10] or modifications of the *tol-pal* pathway of *Escherichia coli, Shigella flexneri*, and *Salmonella enterica* serovar Typhimurium [11], [12] can increase the level of shedding. Especially, deletion of the *tolR* gene in *E. coli* has been shown to result in substantial overproduction of outer membrane particles without loss of membrane integrity [11], [13]. Studies have characterized the protein content of these outer membrane particles [10], [13], and unlike conventional detergent-extracted outer membrane vesicles derived from homogenized bacteria they are almost free of cytoplasmic and inner membrane components and maintain lipoproteins. The outer membrane particles used for those proteomic studies have been derived in small quantities from cells grown to low cell density.

It has been previously proposed that outer membrane particles could be exploited for use as vaccines [10], [12]. The immunogenicity of outer membrane particles from a variety of Gram-negative bacteria has been studied. Consistent with their high content of stimulators of the innate immune system, e.g. lipopolysaccharide (LPS) [7] and Toll-like receptor 2 (TLR2) agonists [14], they are strongly immunogenic in the absence of adjuvant. They have been shown to induce protection in mice against multiple pathogens, including *Salmonella enterica* serovar Typhimurium [15], *Helicobacter pylori* [16], *Vibrio cholera* [17], [18], or to elicit antibodies in mice with *in vitro* bactericidal activity, e.g. for *Neisseria meningitidis* [19]. Recently, outer membrane particles from *Shigella flexneri* 2a have been shown to confer protection in mice after mucosal immunization [20]. Although these studies suggest that outer membrane particles may form the basis of vaccines [15], [17], [18], there remain several problems: their reactogenicity and the difficulty of purifying them in the quantity and at costs that would make them attractive as vaccines for the public sector most impacted by diseases such as shigellosis.

The problem of reactogenicity is amenable to genetic manipulation. A variety of strategies has been examined to attenuate the pyrogenicity of LPS by modifying genes involved in lipid A biosynthesis, e.g. *msbB* and *htrB* in *Shigella* and *E. coli* or *lpxL* in *Neisseria* that are required for complete acylation and thereby pyrogenicity of lipid A [21]–[24]. However, a major remaining difficulty is developing a scalable method for the high volume and low unit cost production of vaccines based on this method.

In this paper we show that high purity outer membrane particles from *Shigella sonnei* mutant strains can be produced from fermentation in chemically defined medium with high yield using a simple purification process thus making production of inexpensive vaccines feasible. We believe that this process will be widely applicable for production of Gram-negative membrane antigens and thus call it the ‘Generalized Modules for Membrane Antigens (GMMA)’ process. In the literature, outer membrane particles that are either naturally released or produced by genetically modified strains are usually referred to as outer membrane vesicles (OMV). The same term has also been used for the vesicles derived by detergent-extraction of homogenized bacteria currently used as vaccines, e.g. MeNZB, an outer membrane vesicle vaccine used to control *Neisseria meningitidis* type B infections in New Zealand. In order to differentiate the two substantially different types of OMV [10] we chose the term GMMA to specify the particles released from the surface of intact cells used in this study.

**Table 1:** Primers used in this study.

*tolR*.Kan.500-5	TCTGGAATCGAACTCTCTCG
*tolR*.Kan.L3	ATTTTGAGACACAACGTGGCTTTCATGGCTTACCCCTTGTTG
*tolR*.Kan.L5	TTCACGAGGCAGACCTCATAAACATCTGCGTTTCCCTTG
*tolR*.Kan.500-3	TTGCTTCTGCTTTAACTCGG
ampli.Kan-5	ATGAGCCATATTCAACGGGAAAC
ampli.Kan-3	TTAGAAAAACTCATCGAGCATCAAA
*galU*.Cm.500-5	AAAATCAACGGTTGCCAGAG
*galU*.Cm.L-3	CGAAGTGATCTTTCCGTCACATTAAATTCTCCTGGACTGTTC
*galU*.ext-5	GCCTGGTGCTTGATATTGC
*galU*.Cm.500-3	GCGCAGGCAAGAGAATGTA
*galU*.Cm.L-5	CAGTTATTGGTGCCCCATCCGTATCGGTGTTATCC
*galU*.ext-3	TCCTGGCTATTGCACAACT
ampli.Cm-5	TGTGACGGAAAGATCACTTCG
ampli.Cm-3	GGGCACCAATAACTGCCTTA
XbaI.*msbB*.5′.F	CTAGTCTAGAAGTGCTTTCAGTGGGTGACG
EcoRV.*msbB*.5′.R	AGCTTGATATCCCATGCTTTTCCAGTTTCGG
EcoRV.*msbB*.3′.F	AGCTTGATATCGGCGAAATCCAACCGTATAAG
XhoI.*msbB*.3′.R	CCGCTCGAGGGGGAAGTTGTTAAGACAGAC
EcoRV.Ery.F	AGCTTGATATCAGAGTGTGTTGATAGTGCAGTATC
EcoRV.Ery.R	AGCTTGATATCACCTCTTTAGCTTCTTGGAAGCT
pS.so53G.oriF	CGTAACCGTAATTACAGCCG
pS.so53G.oriR	GATTTCACCTTACCCATCCC
pS.so53G.wzyF	CGTTGAGGTTTCACGTTTCT
pS.so53G.wzyR	TTACCAATATACCCTCCGCA

## Methods

### Construction of Shigella Sonnei 53G Mutants

*Shigella sonnei* 53G [25] was chosen as parent strain. The null mutants *tolR* [13], *galU* [26], and *msbB1* [21] were obtained by replacing the *gene* coding sequence with a resistance cassette [27]. Kanamycin was used for *tolR*, chloramphenicol for *galU* and erythromycin for *msbB1*. To achieve this, we used a three step PCR protocol to fuse the *gene* upstream and downstream regions to the resistance gene. Briefly, the upstream and downstream regions of the gene were amplified from *Shigella sonnei* 53G genomic DNA with the primer pairs gene.AB.500-5/gene.ABL-3 and gene.AB.L-5/gene.AB.500-3, respectively (details of target ‘gene’, antibiotic cassette ‘AB’ and sequence are reported in Table 1). The kanamycin cassette was amplified from pUC4K [28] and the *cat* gene from pKOBEG [29] using the primers ampli.AB-5/ampliAB-3 (Table 1). Finally the three amplified fragments were fused together by mixing 100 ng of each in a PCR reaction containing the gene.AB.500-5/gene.AB.500-3 primers. The linear fragment to delete *tolR* was used to transform recombination-prone *Shigella sonnei* 53G carrying pAJD434 to obtain the respective deletion mutant *S. sonnei* Δ*tolR*. Recombination-prone *S. sonnei* Δ*tolR* was then transformed with the linear fragment for the deletion of *galU*, resulting in mutant strain *S. sonnei* Δ*tolR* Δ*galU*. A clone of *S. sonnei* Δ*tolR* lacking the virulence plasmid, *S.* *sonnei* –pSS Δ*tolR*, was selected by white appearance on congo red agar. The curing of the virulence plasmid (pSS) was confirmed by the absence of the origin of replication and the plasmid encoded gene *wzy* using primers pS.so53G.oriF/pS.so53G.oriR and pS.so53G.wzyF/pS.so53G.wzyR respectively (Table 1). Two functional *msbB* genes are present in *Shigella* [21]. In the Δ*tolR* background, the copy located on the virulence plasmid (*msbB2*) was removed by curing the plasmid and the plasmid pΔ*msbB*ko::ery was constructed to delete the gene *msbB1* on the chromosome. Upstream and downstream flanking regions of the *msbB1* gene were amplified by PCR with the XbaI.*msbB*.5′.F/EcoRV.*msbB*.5′.R and EcoRV.*msbB*.3′.F/XhoI.*msbB*.3′.R primers, respectively. Both products were cloned into the pBluescript (Stratagene) vector in Max Efficiency® *E. coli* DH5α™^-^T1^R^ (Invitrogen). The *erm* erythromycin resistance gene [30] was amplified with primers EcoRV.Ery.F/EcoRV.Ery.R and was inserted into the EcoRV site between the flanking regions generating pΔ*msbB*ko::ery. Primers XbaI.*msbB*.5′.F/XhoI.*msbB*.3′.R were used to amplify by PCR a linear fragment from pΔ*msbB*ko::ery plasmid, containing the resistance cassette flanked by *msbB1* flanking regions that was used to transform the recombination-prone plasmid-cured *Shigella sonnei* 53G Δ*tolR* strain to generate the *msbB* knockout mutant. Recombination-prone *Shigella sonnei* 53G cells were produced by using the highly proficient homologous recombination system as previously described (*red* operon) [31] encoded on pAJD434 [32]. pAJD434 was subsequently removed from the mutant strains.

### Bacterial Strain Growth Conditions and Media

*Shigella sonnei* and *E. coli* strains were routinely cultured in Luria-Bertani (LB) medium. When required, kanamycin (30 µg/mL), chloramphenicol (20 µg/mL), trimethoprim (100 µg/mL), or ampicillin (100 µg/mL) were added. Tryptic soy agar (30 g/L tryptic soy broth, 15 g/L agar) supplemented with 150 mg/L congo red was used to evaluate the presence of the virulence plasmid in *Shigella*. GMMA were prepared from cultures grown in flasks or in a 5 L fermenter (Applikon) in yeast extract medium (HTMC) or *Shigella sonnei* defined medium (SSDM). HTMC was prepared as follows: yeast extract 30 g/L, KH_2_PO_4_ 5 g/L, K_2_HPO_4_ 20 g/L, MgSO_4_*7H_2_O 1.2 g/L, glycerol 15 g/L, polypropylene glycol (PPG) 0.25 g/L. SSDM was prepared as follows: glycerol 30 g/L, KH_2_PO_4_ 13.3 g/L, (NH_4_)_2_HPO_4_ 4 g/L, MgSO_4_*7H_2_O 1.2 g/L, citric acid 1.7 g/L, CoCl_2_*6H_2_O 2.5 mg/L, MnCl_2_*4H_2_O 15 mg/L, CuCl_2_*2H_2_O 1.5 mg/L, H_3_BO_3_ 3 mg/L, Na_2_MoO_4_*2H_2_O 2.5 mg/L, Zn(CH_3_COO)_2_*2H_2_O 13 mg/L, ferric citrate 2 µM (unless specified differently in text), thiamine 50 mg/L, nicotinic acid 10 mg/L, L-aspartic acid 2.5 g/L, PPG 0.25 g/L. For fermentation, starter cultures were grown from glycerol stocks to OD 0.8 and subsequently transferred to the 5 L fermenter to reach a starting OD of 0.02. Dissolved oxygen was maintained at 30% saturation by controlling agitation and setting maximum aeration. The pH was maintained at 7.2 in HTMC or at 6.7 in SSDM, with 4 M ammonium hydroxide by a pH controller and temperature was kept constant either at 37°C or at 30°C. From flask cultures, supernatants were collected by 10 min centrifugation at 4000 g followed by 0.22 µm filtration or by tangential flow filtration. The optical density (OD) of cultures was measured at 600 nm wavelength.

**Figure 1:**
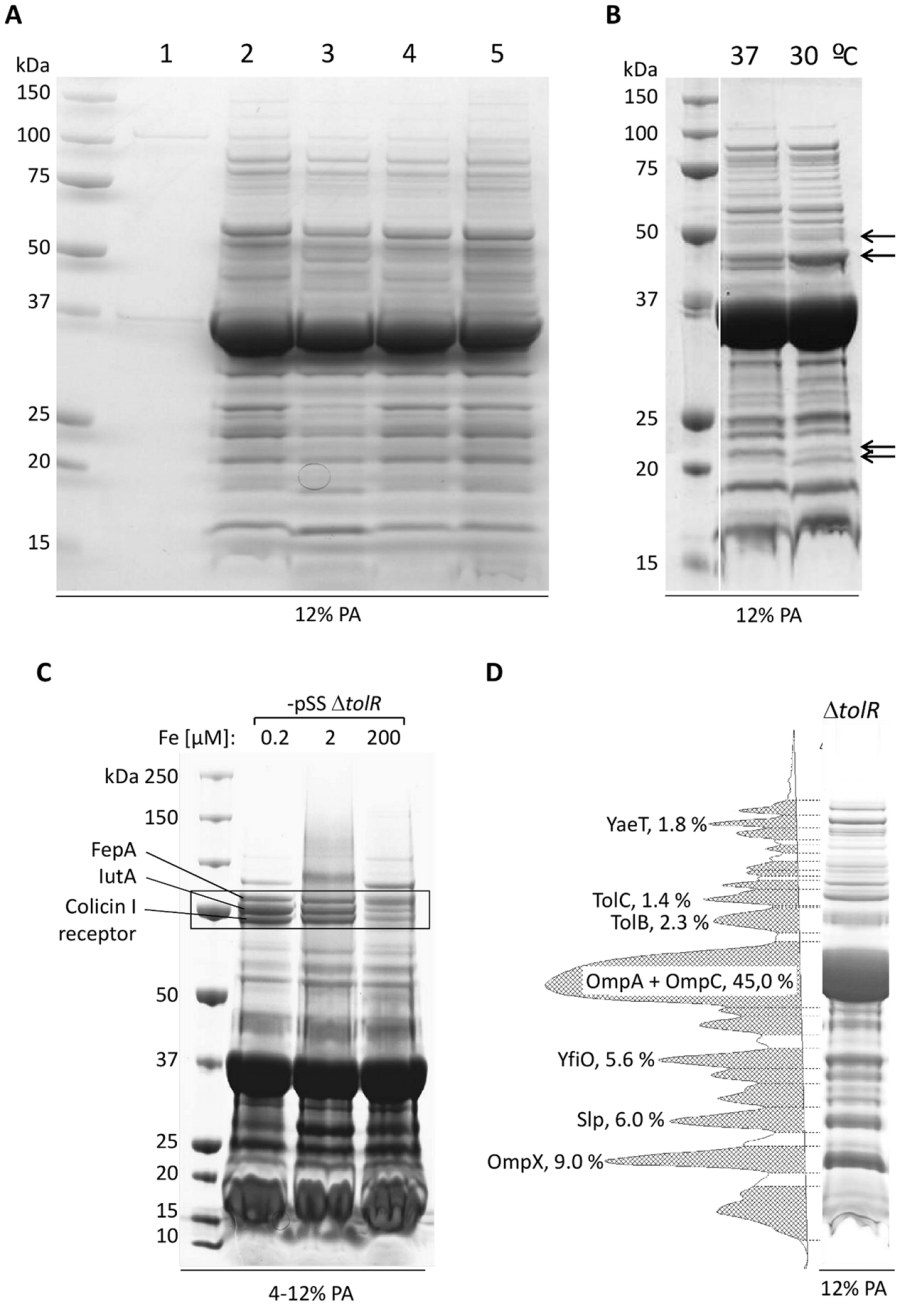
Comparison of S*higella sonnei* GMMA from different strains and different conditions. A)25 ml of culture supernatants were collected from (1) wild type *S. sonnei* 53G, (2) *S. sonnei* Δ*tolR* (3) *S. sonnei* Δ*tolR* Δ*galU*, (4) *S. sonnei* –pSS Δ*tolR*, and (5) *S. sonnei* –pSS Δ*tolR* Δ*msbB* grown in flasks in chemically defined medium at 30°C. Proteins were precipitated from the supernatants and quantified using Bradford assay. 10 µg of samples 2–5, respectively, and the total quantity of sample 1 obtained from 25 mL of supernatant were separated by SDS-PAGE (12% polyacrylamide (PA)). All strains with deletion of the *tolR* gene show an extensive protein profile in the supernatant compared to wild type. B) GMMA were purified by ultracentrifugation from flask cultures of *S. sonnei* –pSS Δ*tolR* grown in chemically defined medium with 100 µM iron at 37°C and 30°C. 10 µg of protein were separated by SDS-PAGE (12% PA). The protein pattern of GMMA obtained at the different temperatures is similar. Visible differences are marked by arrows. C) *S. sonnei* 53G –pSS Δ*tolR* was grown in flasks in chemically defined medium with defined iron concentrations. GMMA were purified by ultracentrifugation and GMMA proteins were separated by SDS-PAGE (4–12% PA). Three bands identified as FepA, IutA, and colicin I receptor were shown to be repressed by high iron concentration. D) Densitometry analysis of GMMA preparation from strain *S. sonnei* Δ*tolR* Δ*galU* grown in a 5 L fermenter to OD 45. The most abundant proteins were identified by protein mass fingerprint and relative amounts were determined by densitometry analysis. Of the highlighted proteins, all proteins with exception of TolB are predicted to be associated with the outer membrane, indicating that approximately 69% of the total protein amount in GMMA is derived from abundant proteins linked to the outer membrane.

### Tangential Flow Filtration Purification

A 2-step tangential flow filtration (TFF) process was used to purify GMMA. During the first TFF step, the culture supernatant which contains the GMMA was separated from the bacteria using a 0.2 µm pore size cassette (Sartocon HYDROSART 0.2 µm, Sartorius). When approximately 80% of the starting feed was recovered as filtrate, the remaining biomass (retentate) was washed in five diafiltration steps with phosphate buffered saline (PBS). The GMMA-containing culture supernatant and the GMMA-containing filtrate of the diafiltration steps were combined. In a modified process the diafiltration of the biomass was omitted. Experiments performed without this diafiltration step are specified in the text. In the second step, the combined filtrate was micro-filtered using a 0.1 µm pore size membrane (Sartocon SLICE 200 0.1 µm, Sartorius) in order to separate GMMA that remain in the retentate from soluble proteins (filtrate). After five diafiltration steps using PBS, the retentate containing the GMMA was collected and sterile filtered using a 0.22 µm Express™ PLUS stericup (Millipore).

### Protein Quantification

Proteins were quantified by Bradford method, using bovine serum albumin as standard. GMMA were boiled for 10 minutes in 3.0 M guanidine hydrochloride prior quantification.

### Negative Staining Electron Microscopy

A drop of 5 µL of GMMA suspension was placed on copper formvar/carbon-coated grids and adsorbed for 5 min. Grids were then washed with few drops of distilled water and blotted with a Whatman filter paper. For negative staining, grids were treated with 2% uranyl acetate in ddH_2_O for 1 min, air-dried and viewed with a CM100 transmission electron microscope (Philips, Eindoven, the Netherlands) operating at 80 kV. Electron micrographs were recorded at a nominal magnification of 60000×.

### Denaturing Mono-Dimensional Electrophoresis

GMMA were denatured for 3 min at 95°C in sodium dodecyl sulfate-polyacrylamide gel electrophoresis (SDS-PAGE) sample buffer containing 2% (wt/vol) SDS. 20 µg of proteins were loaded onto 12% (wt/vol) or 4–12% (wt/vol) polyacrylamide gels (BioRad, Hercules, U.S.A.). Gels were run in 3-(N-morpholino)propanesulfonic acid (MOPS) buffer (BioRad) and were stained with Coomassie Blue R-250.

### Two-Dimensional Electrophoresis

Two hundred micrograms of GMMA were separated by 2-dimentional electrophoresis (2-DE) as previously described [10]. Briefly, proteins were separated in the first dimension on a non linear pH 3–11 gradient and in the second dimension on a linear 4–12% polyacrylamide gradient unless specified in text. Gels were stained with colloidal Coomassie G-250 [33].

**Table 2:** *Shigella sonnei* Δ*tolR* Δ*galU* GMMA-associated proteins identified by proteomics.

A	B	C	D	E
**Outer membrane**				
**1**	*3*	outer membrane channel protein [S. flexneri 2a str. 301]	*tolC*	gi|56480244
**2**	*3*	outer membrane porin protein C [S. sonnei Ss046]	*ompC*	gi|74312736
**3**	*3*	outer membrane protein A [S. sonnei Ss046]	*ompA*	gi|74311514
**4**	*3*	outer membrane protein induced after carbon starvation [S. flexneri 5 str. 8401]	*slp*	gi|110616891
**5**	*3*	outer membrane protein X [S. flexneri 2a str. 301]	*ompX*	gi|56479734
**6**	*2*	outer membrane protein assembly factor YaeT [S. Flexneri 2a str. 301]	*yaeT*	gi|24111612
**7**	*2*	outer membrane protein C [S. boydii CDC 3083-94]	*ompC*	gi|187733369
**8**	*2*	outer membrane receptor FepA [Shigella sonnei Ss046]	*fepA*	gi|74311118
**9**	*2*	ferrichrome outer membrane transporter [Shigella sonnei Ss046]	*fhuA*	gi|74310771
**10**	*2*	colicin I receptor [Shigella sonnei Ss046]	*cirA*	gi|74312677
**11**	*2*	maltoporin [Shigella flexneri 2a str. 301]	*lamB*	gi|56480532
**12**	*4*	putative ferric siderophore receptor [S. sonnei Ss046]	*iutA*	gi|74313972
**13**	*2*	outer membrane protein W [Shigella sonnei Ss046]	*yciD*	gi|74312394
**14**	*2*	serine protease [S. flexneri 2a str. 301]	*sigA*	gi|24114232
**Outer membrane Lipoproteins**				
**15**	*3*	murein lipoprotein [S. flexneri 2a str. 301]	*lpp*	gi|24113066
**16**	*2*	outer membrane lipoprotein LolB [S. flexneri 2a str. 301]	*lolB*	gi|24112608
**17**	*3*	peptidoglycan-associated outer membrane lipoprotein [S. flexneri 2a str. 301]	*pal*	gi|56479690
**18**	*1*	entericidin B membrane lipoprotein [S. flexneri 2a str. 301]	*ecnB*	gi|24115506
**19**	*1*	hypothetical protein S2067 [S. flexneri 2a str. 2457T]	*yedD*	gi|30063370
**20**	*1*	hypothetical protein S4565 [S. flexneri 2a str. 2457T]	*yjeI*	gi|30065519
**21**	*1*	hypothetical protein SF0398 [S. flexneri 2a str. 301]	*ybaY*	gi|24111837
**22**	*1*	RpoE-regulated lipoprotein [S. flexneri 2a str. 301]	*SF2485*	gi|24113773
**23**	*1*	hypothetical protein SSON_2966 [S. sonnei Ss046]	*SSON_2966*	gi|74313380
**24**	*1*	lipoprotein [S. flexneri 2a str. 2457T]	*nlpB*	gi|30063856
**25**	*2*	entry exclusion protein 2 [S. sonnei Ss046]	*exc*	gi|145294038
**26**	*2*	LPS-assembly lipoprotein RplB [S. dysenteriae Sd197]	*rplB*	gi|82775909
**27**	*2*	putative pectinesterase [S. sonnei Ss046]	*ybhC*	gi|74311310
**28**	*4*	outer membrane protein assembly complex subunit YfiO [Shigella sonnei Ss046]	*SSON_2721*	gi|74313154
**29**	*3*	outer membrane lipoprotein [S. flexneri 2a str. 301]	*yraP*	gi|24114441
**30**	*3*	DNA-binding transcriptional activator OsmE [S. flexneri 2a str. 301] lipo	*osmE*	gi|24112862
**31**	*1*	outer membrane protein [S. flexneri 2a str. 301]	*slyB*	gi|24113033
**Periplasmic**				
**32**	*3*	FKBP-type peptidyl-prolyl cis-trans isomerase [S. Flexneri 2a str. 301]	*fkpA*	gi|24114611
**33**	*3*	histidine-binding periplasmic protein of high-affinity histidine transport system [S. sonnei Ss046]	*hisJ*	gi|74312826
**34**	*3*	serine endoprotease [S. flexneri 2a str. 301]	*htrA*	gi|24111599
**35**	*3*	translocation protein TolB [S. flexneri 2a str. 2457T]	*tolB*	gi|30062097
**36**	*1*	molybdate transporter periplasmic protein [S. flexneri 2a str. 301]	*modA*	gi|24111968
**37**	*1*	peptidyl-prolyl cis-trans isomerase A (rotamase A) [S. flexneri 2a str. 301]	*ppiA*	gi|24114628
**38**	*1*	peptidyl-prolyl cis-trans isomerase SurA [S. flexneri 2a str. 301]	*surA*	gi|24111499
**39**	*1*	periplasmic oligopeptide binding protein [S. flexneri 2a str. 2457T]	*oppA*	gi|30062764
**40**	*1*	periplasmic protein [S. flexneri 2a str. 2457T]	*osmY*	gi|30065614
**41**	*2*	arginine 3rd transport system periplasmic binding protein [S. sonnei Ss046]	*artJ*	gi|74311404
**42**	*2*	bifunctional UDP-sugar hydrolase/5′-nucleotidase [S. sonnei Ss046]	*ushA*	gi|74311061
**43**	*2*	cystine transporter subunit [S. sonnei Ss046]	*fliY*	gi|74311733
**44**	*2*	glucan biosynthesis protein G [S. flexneri 5 str. 8401]	*mdoG*	gi|110805056
**45**	*2*	thiosulfate transporter subunit [S. sonnei Ss046]	*cysP*	gi|74312961
**46**	*2*	hypothetical protein SBO_2040 [Shigella boydii Sb227]	*ycdO*	gi|82544504
**47**	*2*	hypothetical protein SFV_2968 [S. flexneri 5 str. 8401]	*yggE*	gi|110806822
**Cytoplasmic**				
**48**	*3*	chaperonin GroEL [S. flexneri 2a str. 301]	*groEL*	gi|24115498
**49**	*3*	dihydrolipoamide dehydrogenase [S. flexneri 2a str. 301]	*lpdA*	gi|56479605
**50**	*1*	purine nucleoside phosphorylase [S. flexneri 2a str. 2457T]	*deoD*	gi|30065622
**51**	*1*	succinyl-CoA synthetase subunit beta [S. flexneri 2a str. 301]	*sucC*	gi|24111996
**52**	*2*	PTS system glucose-specific transporter subunit [S. flexneri 2a str. 301]	*crr*	gi|24113762
**53**	*2*	molecular chaperone DnaK [S. flexneri 2a str. 301]	*dnaK*	gi|24111463
**54**	*1*	pyrroline-5-carboxylate reductase [S. flexneri 2a str. 301]	*proC*	gi|24111764
**55**	*2*	hypothetical protein SF1022 [S. flexneri 2a str. 301]	*SF1022*	gi|24112431
**Inner membrane**				
56	*1*	hypothetical protein SSON_1546 [S. sonnei Ss046]	*ydgA*	gi|74312061
**Unknown**				
**57**	*3*	putative receptor [S. sonnei Ss046]	*SSON_1681*	gi|74312191
**58**	*2*	hypothetical protein SSON_1556 [S. sonnei Ss046]	*ydgH*	gi|74312071
**59**	*2*	hypothetical protein SSON_3340 [S.sonnei Ss046]	*yrbC*	gi|74313729
**60**	*2*	putative lipoprotein [Shigella dysenteriae Sd197]	*ybjP*	gi|82777619
**61**	*1*	hypothetical protein S3269 [S. flexneri 2a str. 2457T]	*ygiW*	gi|30064374

GMMA were purified by 2-step TFF from *S. sonnei* Δ*tolR* Δ*galU* grown in HTMC at 37°C to an OD of 45. GMMA-associated proteins were separated by SDS-PAGE or nano-LC and identified by mass spectrometry. 61 GMMA-associated proteins were identified. The columns show: A) position in list, B) method used to identify each protein (‘1′: identified from total digestion LC/MS-MS, ‘2′: identified from 2D SDS-PAGE PMF, ‘3′ identified from LC/MS-MS and 2D SDS-PAGE PMF, ‘4′ identified from 1D SDS-PAGE PMF), C) annotation, D) gene name, E) accession number. The entries are divided by predicted location. All proteins were analyzed by PSORTb 3.0 and Lipo. If a prediction as lipoprotein was obtained, the protein is listed as lipoprotein irrespective of its PSORTb prediction. Only lipoproteins predicted to be located in the outer membrane have been identified. The other proteins are listed in sections corresponding to their location predicted by PSORTb. For 5 proteins no prediction was obtained by PSORTb or Lipo. These are listed as ‘unknown’.

### Densitometry Analysis

SDS-PAGE and 2-DE gels were scanned with an Image Quant 400 (GE Healthcare). Images were analyzed with the software Image master 2D Platinum 6.0 (Amersham Biosciences).

### In-Gel Protein Digestion and MALDI-TOF Analysis

Protein spots were excised from the gels and processed as previously described [13]. Mass spectra were acquired on a Ultraflex MALDI TOF-TOF mass spectrometer (Bruker Daltonics) in reflectron, positive mode, in the mass range of 900 to 3,500 Da. Spectra were externally calibrated by using a combination of standards pre-spotted on the target (Bruker Daltonics). MS spectra were analyzed by Protein Mass Fingerprint (PMF) with flexAnalysis (flexAnalysis version 2.4, Bruker Daltonics). Monoisotopic peaks were annotated with flexAnalysis default parameters and manually revised. Protein identification was carried from the generated peak list using the Mascot program (Mascot server version 2.2.01, Matrix Science). Mascot was run on a database containing protein sequences deduced from seven sequenced *Shigella* genomes, downloaded from NCBInr or from the Wellcome Trust Sanger Institute database. Genomes used were from strains *Shigella sonnei* 53G, *Shigella flexneri* 2a str. 301, *Shigella flexneri* 2a str. 2457T, *Shigella sonnei* Ss046, *Shigella* boydii Sb227, *Shigella flexneri* 5 str. 8401, *Shigella boydii* CDC 3083-94. Search parameters, mass tolerance, known contaminant ions, validation and handling of multiple matches were performed as described previously [13].

### Protein Precipitation and In-solution Protein Digestion

Proteins from supernatants or purified GMMA were precipitated by adding TCA and deoxycholate to a final concentration of 10% and 0.04%, respectively. The precipitation was allowed to proceed for 30 min at 4°C. The precipitate was recovered by 10 min centrifugation at 20,000×g at 4°C. The pellet was washed once with 10% TCA (wt/vol) and twice with absolute ethanol, dried with Speedvac (Labconco, Kansas City, U.S.A). For analysis by SDS-PAGE, the precipitates were resuspended with 200 mM Tris-HCl, pH 8.8, and quantified. For LC-MS/MS analysis 20 µg of GMMA were precipitated and resuspended in 50 µL, 6 M guanidinium chloride, 5 mM DTT, 200 mM Tris-HCl, pH 8.0. Denaturation proceeded for 60 min at 60°C. Prior to digestion, the solution was diluted 1∶8 with a solution of 100 mM Tris-HCl, pH 8.0, 5 mM DTT and 5 µg of trypsin (Promega) were added to the diluted solution. Digestion was carried out over night at 37°C. The reaction was stopped by adding formic acid to 0.1%. Peptides were extracted using Oasis extraction cartridges (HLB 1cc (30 mg) extraction cartridges, Waters, Milford, MA, USA) and analyzed by LC-*MS/MS*.

### Protein Identification by Nano-LC-MS/MS

Peptides were separated by nano-LC on a NanoAcquity UPLC system (Waters) connected to a Q-ToF Premier ESI mass spectrometer equipped with a nanospray source (Waters). Samples were loaded onto a NanoAcquity 1.7 µm BEH130 C18 column (75 µm×25 mm; Waters) through a NanoAcquity 5 µm Symmetry C18 trap column (180 µm×20 mm; Waters). Peptides were eluted with a 120 min gradient of 2–40% acetonitrile (98%), 0.1% formic acid solution at a flow rate of 250 nL/min. The eluted peptides were subjected to an automated data-dependent acquisition using the MassLynx software, version 4.1 (Waters) where an MS survey scan was used to automatically select multicharged peptides over the m/z ratio range of 300–2000 for further MS/MS fragmentation. Up to eight different peptides were individually subjected to MS/MS fragmentation following each MS survey scan. After data acquisition, individual MS/MS spectra were combined, smoothed, and centroided using ProteinLynx, version 3.5 (Waters) to obtain the peak list file. The Mascot Daemon application (Matrix Science Ltd., London, UK) was used for the automatic submission of data files to in-house licensed Mascot, version 2.2.1, running on a local server. The Mascot search parameters were set to (i) 2 as the number of allowed missed cleavages (only for trypsin digestion), (ii) methionine oxidation as variable modifications, (iii) 0.05 Da as the peptide tolerance, and (iv) 0.05 Da as the MS/MS tolerance. Only significant hits were considered as defined by the Mascot scoring and probability system.

### Bioinformatics

Prediction of protein localization was carried out using PSORTb v3.0 [34] and Lipo program [35].

### Mouse Immunizations

Outbred CD1 mice (female, 4 to 6 weeks of age) received three injections of GMMA via the subcutaneous route on days 0, 21, and 35. Each injection contained GMMA normalized to 0.2 µg or 2 µg of protein and formulated in PBS only, with Freund’s adjuvant (FA), or adsorbed onto aluminum hydroxide (alum), 2 mg/mL, in a final volume of 100 µL. If Freund’s adjuvant was used, Freund’s complete adjuvant (FCA) was used for the first immunization, Freund’s incomplete adjuvant (ICFA) was used for the second and third immunization. Control mice received either adjuvant or PBS alone. Blood samples were collected before immunization and 14 days after the second and third injection. The animal experiments complied with the relevant guidelines of Italy and the institutional policies of Novartis. The animal protocol was approved by the Animal Welfare Body of Novartis Vaccines and Diagnostics, Siena, Italy, approval number AEC 2009-05.

**Figure 2:**
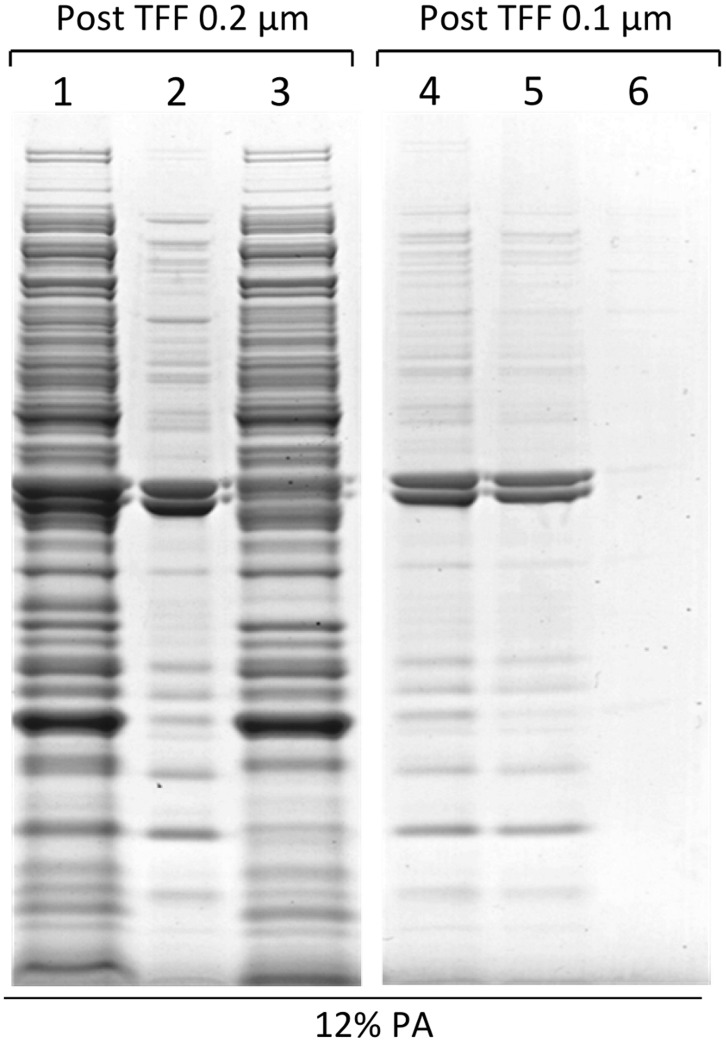
GMMA enrichment and purity after TFF. GMMA were purified from a 5 L fermentation culture of *S. sonnei* Δ*tolR* Δ*galU* grown in HTMC at 37°C to OD 45 using 2-step TFF. In the first step, the culture supernatant which contains the GMMA was separated from the bacteria using a 0.2 µm filter. The biomass was subjected to 5 diafiltration steps and all filtrates were combined with the initial supernatant to obtain the total permeate. To determine the amount of GMMA in the permeate, GMMA were separated from soluble proteins by ultracentrifugation. After ultracentrifugation, the pellet (GMMA) was resuspended in the initial volume of the centrifuged material to normalize all samples to fermentation volume. Equivalent volumes of the 0.2 µm filtrate before ultracentrifugation (1), the resuspended GMMA pellet (2), and the supernatant of the ultracentrifugation (3) were separated by SDS-PAGE (12% PA) and showed a large amount of soluble proteins (3) in comparison to GMMA-associated proteins (2) to be present in the post 0.2 µm TFF permeate. In the second TFF step, GMMA were separated from soluble proteins using a 0.1 µm filter. The retentate (4) was analyzed by ultracentrifugation as described above and was found to contain almost exclusively GMMA (5) as determined by the strong reduction of soluble proteins (6).

**Table 3:** Yield, purity, and recovery rate of GMMA by the high yield production process.

	Fermentation A OD 45	Fermentation B1 OD 30	Fermentation B2 OD 39
**Protein content [mg/L fermentation]**			
**0.2 µm TFF permeate**			
Total protein*	1465	1237	797
GMMA-associated protein	214	143	138
Soluble protein	1251	1094	659
**0.1 µm TFF retentate**			
Total protein#	108	144	118
GMMA-associated protein	120	127	114
Soluble protein	14	5	3
GMMA-associated protein per OD	2.7 mg/L/OD	4.2 mg/L/OD	2.9 mg/L/OD
**Purity of GMMA after 0.1 µm TFF [%]**			
GMMA (GMMA-protein/total protein)	90	88	97
Soluble protein (sol. protein/total protein)	10	3	3
**Recovery of GMMA by 0.1 µm TFF [%]**			
GMMA-protein after 0.1 µm TFF/0.2 µm	56	89	83

* Total protein amount calculated as sum of GMMA-associated protein and soluble protein.

# Total protein amount measured directly by Bradford assay.

*S. sonnei* Δ*tolR* Δ*galU* was grown in HTMC in a 5 L fermenter to high densities of OD 45 (A), OD 30 (B1) and OD 39 (B2) and GMMA were purified using 2-step TFF. Purification from fermentation A was performed including 5 diafiltration steps of the biomass, for GMMA purification from fermentations B1 and B2 the biomass was not subjected to diafiltration. The GMMA content in the permeate of the 0.2 TFF step (culture supernatant) and the retentate of the 0.1 µm TFF (purified GMMA) were determined by separation of GMMA from soluble protein by ultracentrifugation. Protein was quantified using Bradford assay. All samples were normalized to amount per liter fermentation broth. To compare the yields from different ODs, yields are also expressed as amount per liter fermentation per OD.

### Western Blot

GMMA were boiled in loading buffer and loaded on 12% (wt/vol) polyacrylamide-SDS gels (BioRad) or on 2D gels as described. Gels were run in MOPS buffer (BioRad) and protein were subsequently transferred onto nitrocellulose membrane using Trans-blot transfer medium (BioRad). The membranes were blocked in PBS containing 3% (wt/vol) powdered milk, then incubated with mouse polyclonal antisera diluted (1∶1000) in PBS containing 3% (wt/vol) milk for 90 min at 37°C. Membranes were washed three times with PBS containing Tween 20, 0.1% (vol/vol) and then incubated with sheep anti-mouse horseradish peroxidase-conjugated IgG (GE Healthcare, UK Limited), diluted (1∶7500) in PBS containing 3% (wt/vol) milk. Colorimetric staining was performed, after washing the membranes, with SuperSignal West Pico Chemiluminescent Substrate Kit (Pierce, Rockford, U.S.A.) as described by the manufacturer. Positive signals were related to the corresponding proteins by comparing the Western blot membrane to the gel using Ponceau staining of the membrane as a reference and aligning the images with Image master 2D Platinum 6.0.

### Enzyme-linked Immunosorbent Assay (ELISA)

To measure *Shigella sonnei* GMMA-specific immunoglobulin G (IgG) in mice serum, Nunc Maxisorb 96-well plates were coated over night at 2 to 8°C with 100 µL/well of a 0.5 µg/mL suspension of *Shigella sonnei* 53G –pSS Δ*tolR* GMMA, purified from defined medium with 2 µM ferric citrate in the same way as the GMMA in the vaccine, diluted in phosphate-buffered saline (PBS). Plates were then washed three times with 300 µL/well of phosphate-buffered saline containing 0.05% (vol/vol) Tween 20 (PBST) and blocked with PBS containing 1% (wt/vol) BSA for 60 min at 37°C. Serial dilutions of reference and sample sera were prepared in PBST, 1% (wt/vol) BSA in a separate dilution plate, and 100 µL/well of each serial dilution was transferred to the coated plate, incubated for 2 hours at 37°C, and then washed as described above. Bound antibody was detected using a goat anti-mouse IgG conjugated to alkaline phosphatase, diluted in PBST, 1% (wt/vol) BSA to 1∶5000 and incubated for 2 hours at 37°C. After a wash with PBST, 100 µL/well of *p*-nitrophenyl phosphate substrate dissolved in diethanolamine buffer (1 M, pH 9.8) was added, and after 20 minutes optical densities were measured with a plate reader (ELx800, BioTek) at 405 and 490 nm wavelength. Absorbance at 490 nm was subtracted from the absorbance at 405 nm. Results are expressed in arbitrary ELISA units relative to a standard serum raised against GMMA from *S. sonnei* 53G Δ*tolR* Δ*galU*. One unit equals the reciprocal of the dilution of the standard serum giving an OD_405–490_ of 1 in the assay. All samples were measured in duplicate.

### Statistical Analysis

Antibody levels (ELISA units) in different groups after the third immunization were compared by non-parametric Kruskal-Wallis and Mann-Whitney tests. A p value of 0.05 was considered to be significant. For multiple comparisons the p value considered to be significant in each of the comparisons was adjusted according to the number of analyses.

**Figure 3:**
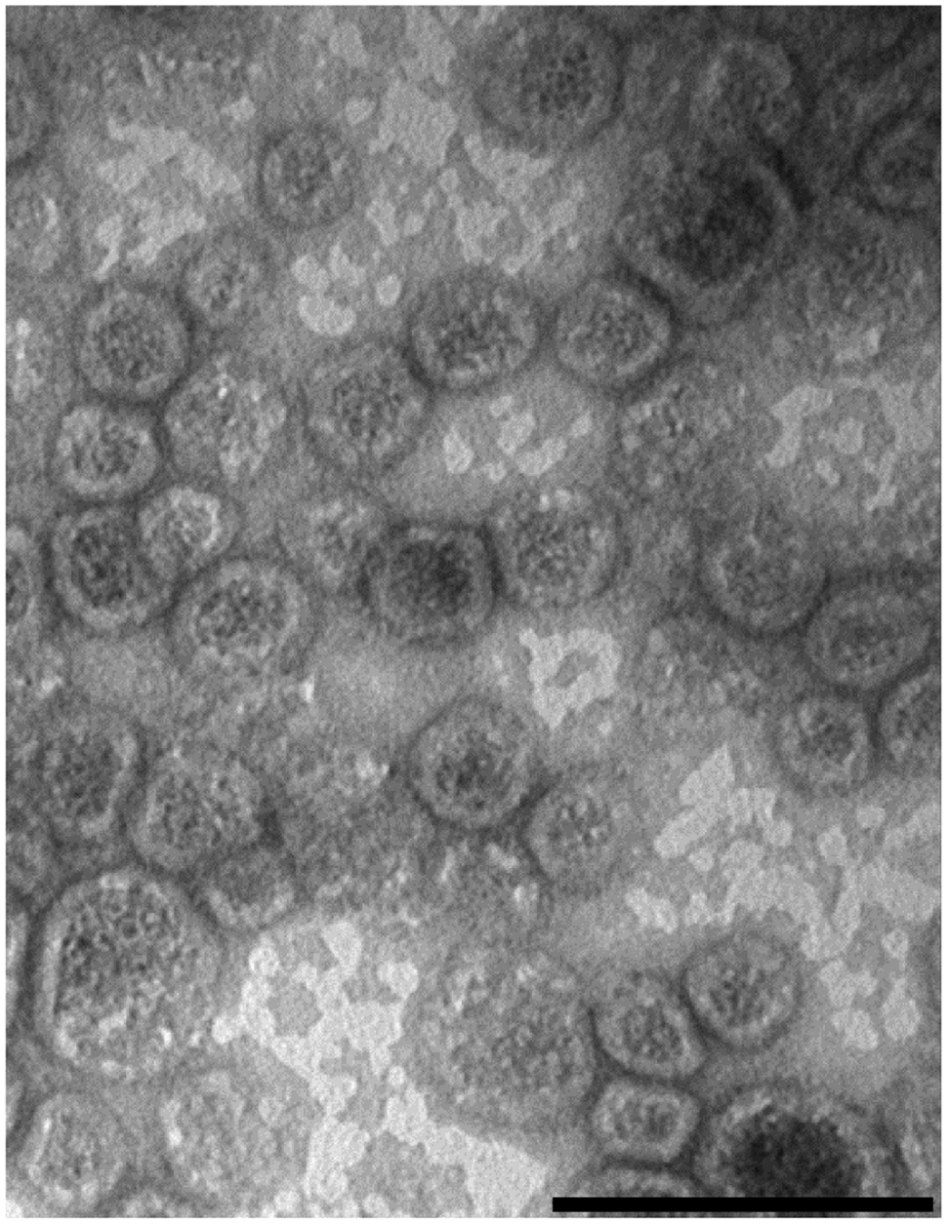
Electron microscopy of *Shigella sonnei* Δ*tolR* Δ*galU* GMMA. GMMA were isolated from the culture supernatant of *S. sonnei* –pSS Δ*tolR* Δ*msbB* by TFF, prepared for negative staining, and viewed by electron microscopy revealing the presence of well-organized membrane vesicles with a diameter of about 30–60 nm. Bar length  = 100 nm.

**Figure 4:**
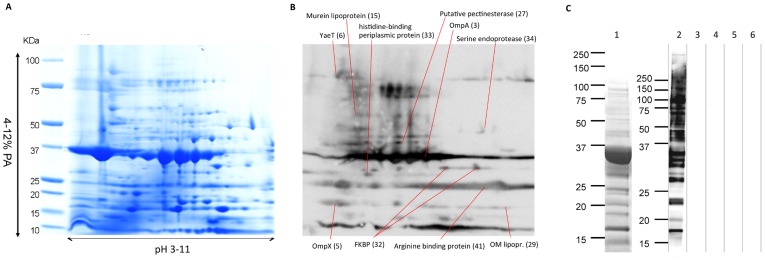
2D gel electrophoresis of *Shigella sonnei* Δ*tolR* Δ*galU* GMMA and immunoblot. A)200 µg of proteins from *S. sonnei* Δ*tolR* Δ*galU* GMMA were separated in the first dimension on a non linear pH 3–11 gradient, and in the second dimension on a 4–12% polyacrylamide gradient. Visible bands were identified by protein mass fingerprint. OmpA and OmpC were quantified with Image master 2D Platinum 6.0. B) Sera from mice immunized with GMMA from *S. sonnei* Δ*tolR* Δ*galU* were used to study the subset of proteins present in GMMA that are able to raise antibodies. A 2D gel containing 20 µg of GMMA protein from *S. sonnei* Δ*tolR* Δ*galU* was blotted and the membrane was incubated with sera from immunized mice with GMMA from *S. sonnei* Δ*tolR* Δ*galU* in combination with Freund’s adjuvant. Several reactive proteins were identified. The numbers behind the names refer to the position of the proteins in Table 2. C) To verify that the signal observed in the 2D Western blot was due exclusively to antibody raised upon immunization with GMMA, 10 µg of GMMA were separated by 1D SDS-PAGE (12% PA) and stained with Coomassie (1) or transferred to a membrane. Western blots were developed using (2) sera raised against GMMA from *S. sonnei* Δ*tolR* Δ*galU* as used for the 2D Western blot in B, (3) preimmune serum, (4) sera raised in mice immunized with Freund’s adjuvant or (5) PBS, or (6) secondary antibody only. A signal could only be observed when sera raised against GMMA were used (2).

**Figure 5:**
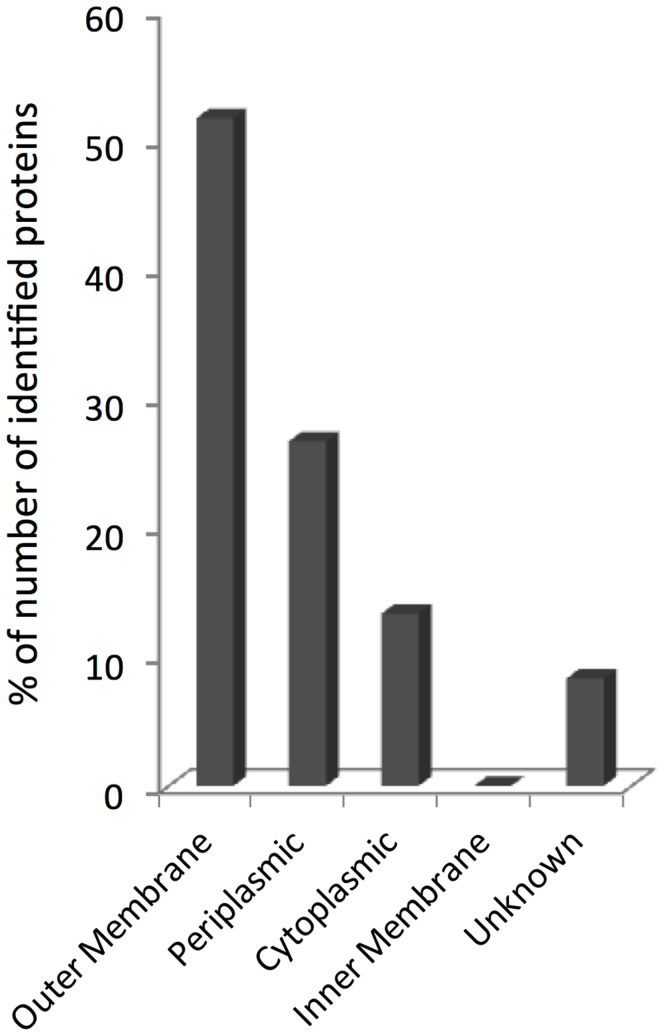
*Shigella sonnei* Δ*tolR* Δ*galU* GMMA proteome. The 61 GMMA-associated proteins that were identified are grouped into families based on their predicted cellular location, according to bioinformatic prediction by PSORTb v3.0 [34] and Lipo program [35]. The ‘outer membrane’ column comprises outer membrane proteins (identified by PSORTb) and lipoproteins predicted to be located in the outer membrane (identified by Lipo). No lipoproteins associated with the inner membrane were identified. The distribution shown is based on the number of identified proteins predicted to be located in a certain compartment. It does not reflect the protein amount. As analyzed by densitometry (Fig. 1D), the outer membrane fraction contains at least 69% of the total protein present in GMMA.

## Results

### Generation of a Shigella Sonnei 53G Strain Capable of Overproducing Modified GMMA

The first aim of the study was to investigate if *Shigella sonnei* 53G could be developed as a strain suitable to overproduce GMMA through modification of the Tol-Pal system. A null mutation of the *tolR* gene was introduced as this has previously been demonstrated to result in overproduction of GMMA in *E. coli* [11], [13]. The mutation in the *tolR* gene led to the release of large amounts of GMMA from the surface of *S. sonnei* 53G as assessed by SDS page (Fig. 1A). The deletion of *tolR* had no detectable influence on bacterial growth (data not shown). In addition, to test if GMMA overproduction is also feasible in strains with additional genetic modifications we removed the O antigen of the LPS, either by deletion of *galU* [26] or by curing the virulence plasmid from strain *S. sonnei* 53G Δ*tolR* as the biosynthesis genes for the O antigen in *Shigella sonnei* are encoded on the plasmid [36]. GMMA obtained from *S. sonnei* Δ*tolR* Δ*galU* showed a similar protein profile to GMMA obtained from *S. sonnei* Δ*tolR* with minor differences in the 37 kDa to 50 kDa range and proteins smaller than 30 kDa appeared to be less abundant in *S. sonnei* Δ*tolR* Δ*galU* (Fig. 1A). Also GMMA obtained from the plasmid-cured *S. sonnei* Δ*tolR* mutant (*S. sonnei* –pSS Δ*tolR*) showed a nearly identical protein pattern to GMMA from *S. sonnei* Δ*tolR* (Fig. 1A).

Furthermore, the genes *msbB1* and *msbB2* involved in lipid A biosynthesis were deleted since these deletions have previously been reported to decrease LPS toxicity in *Shigella* [21]. As the gene *msbB2* is encoded on the virulence plasmid and thus absent in *S.* *sonnei* –pSS Δ*tolR* we deleted the chromosomal gene *msbB1* in this strain to generate a mutant strain lacking *msbB1* and *msbB2*. For simplicity the Δ*msbB1*Δ*msbB2* mutant is referred to as the Δ*msbB* mutant. The Δ*msbB* mutant was selected at 37°C on LB plates and grew in LB and yeast extract at 37°C with a duplication time of about 55 min compared to a duplication time of about 28 min for the single Δ*tolR* mutant. In the defined medium developed for fermentation, the plasmid-cured Δ*tolR* Δ*msbB* mutant strain (*S.* *sonnei* –pSS Δ*tolR* Δ*msbB*) was able to grow to high optical density (OD) at 30°C, but grew poorly at 37°C. Thus, for generation of GMMA from *S. sonnei* –pSS Δ*tolR* Δ*msbB* cultivation in chemically defined medium at a growth temperature of 30°C was chosen. GMMA from *S. sonnei* –pSS Δ*tolR* Δ*msbB* produced under these conditions showed a similar protein pattern to GMMA generated by *S. sonnei* –pSS Δ*tolR* and *S. sonnei* Δ*tolR* with only minor variation in relative amounts of proteins visible by SDS-PAGE in the 45–75 kDa range (Fig. 1A). In order to test if the lower temperature would change the GMMA composition we compared GMMA derived from *S. sonnei* –pSS Δ*tolR* at 30°C or 37°C. Only few differences were detected as highlighted in Fig. 1B, indicating that GMMA can be generated at 30°C without major effects on the composition. In conclusion, deletion of *tolR* greatly enhanced GMMA release while additional genetic modification of the strain or a change in growth temperature only had minor effects on the protein composition visible by SDS-PAGE.

### High Density Cultivation of Shigella Sonnei

To investigate the feasibility to produce GMMA at large scale, *S. sonnei* 53G Δ*tolR* Δ*galU*, *S. sonnei* 53G –pSS Δ*tolR*, and *S. sonnei* 53G –pSS Δ*tolR* Δ*msbB* were tested for their capacity to grow to high densities in a 5 liter reactor. Starter cultures were grown in flasks to OD 0.8 and were then transferred to the 5 L fermenter to reach a starting OD of 0.02. Dissolved oxygen was maintained at 30% saturation. The pH was maintained at 7.2 in HTMC or at 6.7 in SSDM and the temperature was kept constant either at 37°C or at 30°C when *S. sonnei* 53G –pSS Δ*tolR* Δ*msbB* was used. Under these conditions, cultures with optical densities of 45 to 80 were obtained.

Iron-regulated proteins have previously been shown to be important in vaccine formulations against *Pasteurella* and *Salmonella* [37], [38]. Thus, we evaluated if the GMMA process would allow the upregulation of iron-regulated proteins. Growth of *S.* *sonnei* 53G –pSS Δ*tolR* with 0.2 µM iron concentration in chemically defined medium led to the induction of iron-regulated proteins but hindered high density cultivation of bacteria. The addition of 2 µM iron to the medium was sufficient to allow optimal growth and the induction of three iron-regulated proteins visible by SDS-PAGE (Fig. 1C), identified by protein mass fingerprint as FepA (gi|74311118), IutA (gi|74313972) and Colicin I receptor (gi|74312677). The expression of these proteins was reduced when bacteria were grown in 200 µM iron (Fig. 1C). In bacteria grown in HTMC the iron-regulated proteins are expressed to a similar level as in chemically defined medium with 200 µM iron (data not shown). Their presence was confirmed by protein mass fingerprint analysis of GMMA generated from *S. sonnei* Δ*tolR* Δ*galU* grown in HTMC (Table 2, proteins 8, 10, 12). Growth of *S.* *sonnei* 53G –pSS Δ*tolR* Δ*msbB* at 30°C in defined medium with 2 µM iron also enhanced expression of FepA and IutA. Colicin I receptor (marked in Fig. 1C) was less expressed than in GMMA from *S. sonnei* 53G –pSS Δ*tolR* prepared from cultures grown at 37°C (data not shown).

### Purification of GMMA from High Density Culture Supernatant

So far, GMMA have always been purified from flask cultures by ultracentrifugation [13]. Cultures were centrifuged at low speed (4000 g) to separate biomass from supernatant which was subsequently filtered through a 0.22 µm filter. GMMA present in the supernatant were collected by ultracentrifugation, washed, and then resuspended and stored in PBS [10], [13]. Since this technique is not suitable for large volumes we developed a scalable purification method to purify GMMA from high density cultures using tangential flow filtration (TFF). In TFF, also known as crossflow filtration, the feed stream is pumped tangentially across the surface of the membrane rather than into the filter as in conventional ‘dead-end’ filtration. A proportion of the soluble components and particles smaller than the membrane’s pores penetrates the filter (filtrate/permeate). The remainder (retentate) is circulated back to the reservoir and over the filter again. In this way, the larger particles do not build up at the surface of the filter but are swept away by the tangential flow allowing smaller molecules to continuously reach and pass through the membrane. This feature makes TFF an efficient process for size separation, concentration and diafiltration.

GMMA were purified from fermentation cultures in a 2-step TFF process. In the first step, the culture supernatant that contains the GMMA was separated from the bacteria using a 0.2 µm filter. In this step, the bacteria remained in the retentate and GMMA transferred into the filtrate. In the second filtration step using a 0.1 µm filter, GMMA were separated from soluble components present in the culture supernatant, including proteins secreted by the bacteria or released by lysis. In this step, GMMA were retained by the filter and collected and concentrated in the retentate whereas soluble proteins passed through the filter. We tested this purification process under two slightly different conditions. Firstly, when the fermentation culture of *S. sonnei* Δ*tolR* Δ*galU* reached OD 45, the culture was transferred directly from the fermenter to the first TFF and the culture supernatant containing the GMMA was collected. The retained biomass was washed with 5 volumes of PBS buffer (diafiltration) to recover remaining GMMA and the filtrate containing these GMMA was combined with the culture supernatant. In the slightly modified purification process tested, this diafiltration step was omitted. Proteins were quantified by Bradford method. In the purification performed with diafiltration of the biomass the total protein content of the TFF 0.2 µm filtrate was approximately 1.5 g/L of fermentation culture of which 15% was GMMA-associated as determined by separation of the soluble components from the high molecular weight portion (GMMA) via an ultracentrifuge step (Fig. 2 and Table 3). In the second TFF step, GMMA were concentrated in the retentate and washed with five volumes of PBS to remove remaining soluble proteins. As TFF is usually performed under non-sterile conditions, the final retentate was sterilized by filtration through a 0.22 µm filter. An aliquot of the sterilized retentate was subjected to ultracentrifugation to determine the content of GMMA as above. As shown in Fig. 2 most of the proteins in the retentate are GMMA-associated. Protein quantification of the retentate, of the GMMA fraction, and of the supernatant of the ultracentrifugation step (soluble proteins) determined that 90% of all protein present in the retentate was GMMA-associated (Table 3). Thus, soluble proteins were efficiently removed in this step (Fig. 2). GMMA recovery after the 0.1 µm TFF step was 56% of the quantity present after the 0.2 µm cassette. The final yield of GMMA was 120 milligrams of proteins per liter of fermentation (Table 3). In two subsequent tests of the purification method with *S. sonnei* Δ*tolR* Δ*galU* without diafiltration of the biomass, a lower amount of GMMA was obtained in the 0.2 µm TFF filtrate (Table 3). However, recovery of GMMA in the second TFF step (0.1 µm) was enhanced resulting in an overall similar yield of GMMA with equivalent purity (Table 3 and suppl. figures Fig. S1, Fig. S2). This suggests, firstly, that washing of the biomass increases the recovery GMMA from the fermentation culture, and secondly, that a higher starting concentration might be beneficial for the second TFF step. The 0.1 µm TFF step can likely be optimized to take advantage of the larger amounts of GMMA obtained by diafiltration of the biomass. Fermentations of *S. sonnei* –pSS Δ*tolR* Δ*msbB* resulted in yields of 140 mg/L from a culture at OD 65 (2.2 mg/L/OD) and 230 mg/L from a culture at OD 80 (2.9 mg/L/OD), demonstrating that the yield of GMMA can be further improved by growing the culture to a higher OD.

The preparation of GMMA generated from *S. sonnei* –pSS Δ*tolR* Δ*msbB* obtained after the second TFF step was subjected to electron microscopy analysis, revealing the presence of well-organized membrane vesicles with a diameter of about 30–60 nm (Fig. 3) which is consistent with the reported average size of 40±20 nm of outer membrane particles produced by *E. coli tol-pal* mutants [11].

### Characterization of GMMA Protein Content

GMMA purified by TFF from *S. sonnei* 53G Δ*tolR* Δ*galU* grown in high density culture were characterized to confirm their integrity and to analyze their protein content. One- and two-dimensional SDS-PAGE of GMMA and densitometry analysis (Fig. 1D and Fig. 4) were used to determine the protein profile and to study relative protein quantities of the most abundant proteins. Most of the Coomassie blue-stained bands and spots were identified using peptide mass fingerprint (Table 2). OmpA and OmpC are known to be among the most abundant proteins present in the outer membrane. In fact, densitometry analysis of GMMA from *S. sonnei* Δ*tolR* Δ*galU* grown in HTMC and analyzed by 1D SDS-PAGE indicated that OmpA and OmpC together contribute for 45% of the total protein; OmpX, 9%; Slp, 6%; YfiO, 5.6%; TolB, 2.3%; TolC 1.4%; and YaeT, 1.8% (Fig. 1D). With the exception of the predicted periplasmic protein TolB, all of these proteins are predicted to be associated with the outer membrane. YfiO is predicted to be an outer membrane lipoprotein. OmpA, OmpC, OmpX, Slp, TolC, and YaeT are predicted to be outer membrane proteins. Thus, the seven most abundant outer membrane-associated proteins account for approximately 69% of the protein amount in GMMA. Further densitometry analysis after 2D SDS-PAGE determined that there are approximately equal quantities of OmpA and OmpC (OmpA:OmpC is 1∶0.83 by densitometry of a Coomassie blue-stained gel). In order to identify the diverse and less expressed proteins, GMMA were studied by proteolytic digestion and reverse phase liquid chromatography coupled to MS/MS. 61 proteins were identified in total (LC-MS/MS, 1D and 2D SDS-PAGE PMF) (Table 2), with 31 of these proteins predicted to be associated with the outer membrane (Fig. 5). Of these, 14 proteins were predicted to be outer membrane proteins and 17 to be outer membrane lipoproteins. In addition, 16 proteins were predicted to be periplasmic, 8 to be cytoplasmic, 1 to be located in the inner membrane, and for 5 proteins no prediction could be obtained. No inner membrane lipoproteins were predicted. Thus, GMMA generated by the high yield production process are mostly composed of outer membrane-associated and periplasmic proteins as previously seen for outer membrane particles release from cultures at the early logarithmic phase [10], [13].

### GMMA Immunogenicity

**Figure 6:**
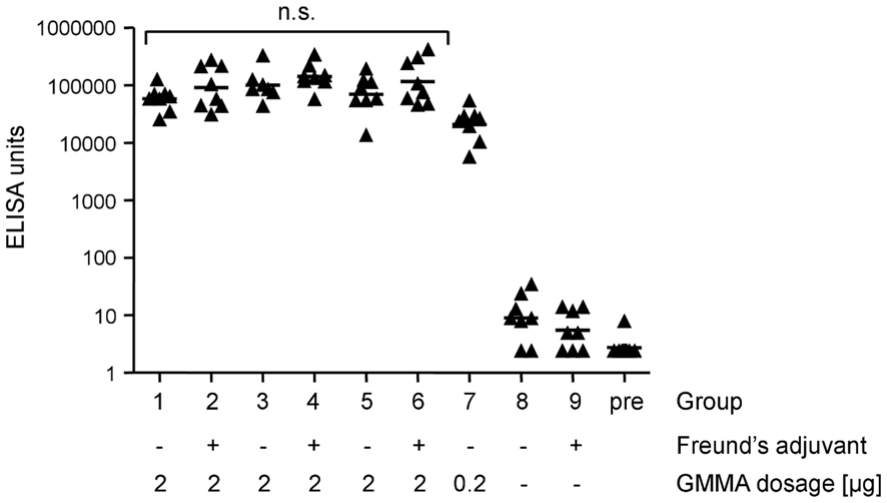
ELISA analysis of sera reactivity against GMMA. Groups 1–6 received 2 µg of GMMA with or without Freund’s adjuvant (FA), group 1) GMMA from *S. sonnei* Δ*tolR* Δ*galU* (grown in HTMC, 37°C), 2) GMMA of group 1 plus FA, 3) GMMA *S. sonnei* –pSS Δ*tolR* (defined medium, 37°C), 4) GMMA of group 3 plus FA, 5) GMMA from *S. sonnei* –pSS Δ*tolR* Δ*msbB* (defined medium, 30°C), 6) GMMA of group 5 plus FA. Group 7 received 0.2 µg of GMMA from *S. sonnei* –pSS Δ*tolR* Δ*msbB*. Control groups were immunized with PBS alone (group 8) or FA alone (group 9). Sera from individual mice obtained 14 days after the third immunization and pooled preimmune sera from each group respectively were assayed in dilutions of 1∶1000, 1∶10,000, and 1∶100,000 on GMMA from *S. sonnei* 53G –pSS Δ*tolR* as coating and arbitrary units were calculated. Data are presented as scatter plots of ELISA units determined in individual mice (groups 1–9) or of the pooled preimmune sera (pre). The horizontal lines represent the geometric mean. ELISA units of groups 1–6 receiving 2 µg of GMMA were analyzed using the non-parametric Kruskal-Wallis test to compare the immunogenicity of the different GMMA to each other and with and without FA. No statistically significant differences were found (n.s.). Reduction of the immunization dosage of *S. sonnei* –pSS Δ*tolR* Δ*msbB* GMMA to 0.2 µg (group 7) resulted in statistically significant reduction of ELISA units in the sera of the immunized animals compared to sera of mice immunized with 2 µg of the same GMMA (group 5) as determined by Mann-Whitney test (p = 0.0047). All groups receiving GMMA showed higher *S. sonnei* –pSS Δ*tolR*-specific antibody responses than groups immunized with PBS or FA alone (Mann-Whitney, p≤0.003). For all comparisons a p value smaller than 0.05 was considered to be significant.

Groups of 8 CD1 mice were immunized 3 times with GMMA (2 µg of total protein) obtained from *S. sonnei* 53G –pSS Δ*tolR* and *S. sonnei* 53G –pSS Δ*tolR* Δ*msbB*, both grown in defined medium with 2 µM iron, and *S. sonnei* 53G Δ*tolR* Δ*galU* grown in HTMC. GMMA from *S. sonnei* 53G –pSS Δ*tolR* Δ*galU*, *S. sonnei* 53G –pSS Δ*tolR* and *S. sonnei* 53G –pSS Δ*tolR* Δ*msbB* were also administered in combination with Freund’s adjuvant (FA). Freund’s complete adjuvant was used in the first immunization and Freund’s incomplete adjuvant was used in the second and third immunization. In addition, a lower dosage of 0.2 µg of GMMA from *S.* *sonnei* 53G –pSS Δ*tolR* Δ*msbB* was tested. Serum samples were obtained 2 weeks after the second and third doses and analyzed individually. Mice immunized with GMMA showed very high IgG responses to all 3 types of GMMA that were tested. No difference was found between groups immunized with different GMMA or between groups receiving the same GMMA with or without FA (Fig. 6). Adsorption of GMMA onto alum as adjuvant also did not have an effect on the IgG response (data not shown). Control mice immunized with PBS or FA alone had very low levels of anti-GMMA antibodies (Fig. 6). The 10-fold lower dosage of GMMA from *S. sonnei* 53G –pSS Δ*tolR* Δ*msbB* (0.2 µg) resulted in a statistically significant, approximately 3-fold reduction in the IgG response compared to the group immunized with 2 µg of the same GMMA. However, the IgG response to the lower dosage still showed an approximately 8000-fold increase compared to preimmune sera (Fig. 6).

To investigate which components of GMMA were responsible for the reactivity of the sera, 2D Western blots were performed. As GMMA from *S. sonnei* 53G Δ*tolR* Δ*galU* were characterized best in respect to their protein content, sera from mice immunized with the GMMA from *S. sonnei* Δ*tolR* Δ*galU* were used to probe blots of 2D SDS-PAGE of GMMA from the same strain. Reactive proteins were identified by protein mass fingerprint. Several proteins were detected by the sera (Fig. 4 B) of which OmpA gave the strongest response. OmpC which is as abundant in GMMA as OmpA was not detected. Not all of the visible reactive proteins could be identified.

## Discussion

Recent advances in genomics and reverse vaccinology have identified promising protein targets for vaccines [39]. In many cases, suitable candidate antigens for Gram-negative bacterial vaccines are outer membrane proteins and these pose particular challenges in their expression and purification and in serotype variability. An ideal delivery system especially for bacterial vaccines for developing countries will encompass multiple antigens and enable vaccines to be rapidly tailored to local and changing antigenic serotypes. Ideally, it will also be inexpensive to manufacture. We propose a platform for rapid development and delivery of vaccines against Gram-negative bacteria. The approach is based on the production of outer membrane particles we have named GMMA by genetically modified bacteria. Using genetic manipulation, it is possible to increase their yield, to remove immunodominant structures, to overexpress certain antigens, and to reduce the endotoxic activity [10], [13], [19], [21], [26], [40], [41]. GMMA could potentially be a safe, effective and low cost vaccine but need a practical way of manufacture at scale.

*Shigella sonnei* 53G was chosen for a first approach to develop a scalable process and a null mutation of the *tolR* gene was introduced to overproduce GMMA as previously described for *E. coli* [13]. To verify that the process is applicable to produce GMMA harboring modified lipid A, which would be more suitable for use as vaccine, and/or lacking the O antigen of the LPS we grew high density cultures of *S. sonnei* Δ*tolR* Δ*galU, S. sonnei* –pSS Δ*tolR* (cured of the virulence plasmid pSS), and *S. sonnei* –pSS Δ*tolR* Δ*msbB* in a 5 L fermenter in complex (HTMC) or chemically defined medium. Chemically defined medium was used to avoid contamination from proteins present in complex media and to have the possibility to regulate iron concentration.

Bacteria were removed from the culture supernatant by a tangential flow filtration step using a 0.2 µm membrane. A second tangential flow filtration step with a 0.1 µm membrane was used to concentrate GMMA and to remove soluble proteins. This choice of appropriate molecular weight membranes allowed the purification of GMMA in an easy, efficient, and scalable process. After purification, approximately 90% of all protein was consistently GMMA-associated with reproducible yields of more than 100 mg of GMMA-associated protein per liter fermentation volume from OD 30–45 cultures of *S. sonnei* Δ*tolR* Δ*galU*. The integrity of GMMA obtained by this process was confirmed using electron microscopy. The purity and yield can likely be increased as indicated by fermentations with *S. sonnei* –pSS Δ*tolR* Δ*msbB* to densities of 65 and 80. Furthermore, first results obtained by quantitative amino acid analysis of different types of GMMA indicated an at least two-fold higher protein amount in the GMMA preparations than determined by the Bradford assay used in this study (data not shown). Still, assuming an average yield of 100 mg/L fermentation and a dosage of 25 µg as used for the MeNZB outer membrane vesicle meningococcal vaccine, at least 400,000 doses could be obtained from a 100 L fermenter.

A proteomic approach confirmed that *Shigella sonnei* 53G Δ*tolR* Δ*galU*-derived GMMA are composed mostly of outer membrane and periplasmic components. They conserve lipophilic polypeptides. Only a small number of cytoplasmic components and one inner membrane protein were predicted. Thus, the proteomic analysis of GMMA obtained from an OD 45 culture revealed a similar composition as previously seen in proteomic analyses of outer membrane particles that were obtained from cultures at early logarithmic phase to avoid impurities by cytoplasmic proteins [10], [13].

In accordance with previous reports [15], [17] GMMA were highly immunogenic in mice with titers around 1∶100,000 after administration of 2 µg of GMMA with and without adjuvant. A 10-fold lower dosage of GMMA (without adjuvant) resulted in only a 3-fold reduction and still very high antibody titers suggesting that low amounts of GMMA might be sufficient for vaccination. GMMA from the *msbB* mutant *S. sonnei* strain did not show a difference in immunogenicity which was expected due to a recent report that the resulting lipid A modification does not affect LPS recognition in mice [42]. Immunoblots confirmed that antibodies to proteins, including outer membrane proteins OmpA, OmpX, and YaeT, strongly contributed to the reactivity of the sera. Interestingly, the outer membrane protein OmpC which represents about 20% of protein in GMMA was not detected by sera raised against GMMA. Previously, an immunoproteomic analysis of isolated outer membrane proteins of *Shigella flexneri* 2a [43] also failed to detect OmpC as immunogenic protein. This could suggest that either OmpC is not immunogenic or that epitopes potentially recognized by antibodies are not maintained after SDS-PAGE. This might also apply to other membrane proteins that were not found by the Western blot analysis even though not all reactive proteins could be identified.

The *msbB* mutant strain of *Shigella* lacking the genes *msbB1* and *msbB2* [21] was generated to investigate if the production process was applicable to GMMA with modified lipid A. A previous report [21] had shown that these deletions result in the synthesis of a penta-acylated lipid A instead of a hexa-acylated lipid A in *Shigella* [21]. While the *S. sonnei* –pSS Δ*tolR* Δ*msbB* mutant grows in rich media at 37°C temperature, its growth is impaired in the chemically defined medium developed for fermentation at 37°C but shows a normal growth in this medium at 30°C. Previously, a *Shigella flexneri* 5a *msbB* mutant and an *E. coli msbB* mutant in the K-12 background were reported not to show any growth defects [21], [23]. In contrast, an *msbB* mutant of the clinical isolate *E. coli* H16 formed filaments when grown at 37°C but not at 30°C or when functionally complemented by the cloned *msbB* gene [44]. The *S. sonnei* –pSS Δ*tolR* Δ*msbB* mutant strain used in this study does not form filaments. The reason for the slower growth at 37°C, especially in defined medium, is not clear and could be a result of the background of the strain, the combination of the *tolR* and *msbB* mutation, or a suboptimal composition of the defined medium that can likely be optimized. Importantly, a comparison of the protein pattern of GMMA generated from *S. sonnei* –pSS Δ*tolR* at 37°C and 30°C showed only minor differences in the protein profile visible by SDS-PAGE indicating that the change in temperature does not have major effects on GMMA composition.

In summary, we have identified an easy process to produce large quantities of GMMA from high density culture. GMMA purified from fermentation are extremely pure particles composed almost exclusively of outer membrane and periplasmic components. The simplicity and high yield of the process support its applicability for large scale manufacturing. We have also shown that this process can be used with strains genetically modified to reduce reactogenicity or to remove immunodominant antigens, e.g. the O antigen. While this work focused on *Shigella sonnei*, we believe that this technology is an innovative platform for efficient vaccine manufacturing for Gram-negative bacteria.

## Supporting Information

Figure S1 **GMMA enrichment and purity after TFF without diafiltration of the biomass.** GMMA were purified from a 5 L fermentation culture of *S. sonnei* Δ*tolR* Δ*galU* grown in HTMC at 37°C to OD 39 (fermentation B2 in Table 3) using 2-step TFF. In the first step, the culture supernatant which contains the GMMA was separated from the bacteria using a 0.2 µm filter without further diafiltration of the biomass. To determine the amount of GMMA in the permeate GMMA were separated from soluble proteins by ultracentrifugation. After ultracentrifugation, the pellet (GMMA) was resuspended in the initial volume of the centrifuged material to normalize all samples to fermentation volume. Equivalent volumes of the 0.2 µm filtrate before ultracentrifugation (1), the resuspended GMMA pellet (2), and the supernatant of the ultracentrifugation (3) were separated by SDS-PAGE (12% PA) and showed a large amount of soluble proteins (3) in comparison to GMMA-associated proteins (2) to be present in the post 0.2 µm TFF permeate. In the second TFF step, GMMA were separated from soluble proteins using a 0.1 µm filter. The retentate (4) was analyzed by ultracentrifugation as described above and was found to contain almost exclusively GMMA (5) as determined by the strong reduction of soluble proteins (6). The high recovery rate of 83% in this process (see Table 3) is reflected in the similar strength of the visible protein bands in lane 2 (GMMA in the 0.2 µm TFF filtrate) and lane 5 (GMMA in the 0.1 µm retentate).
(TIF)

Figure S2 **Reproducibility of purity and protein composition of GMMA obtained by the high yield production process.** *S. sonnei* Δ*tolR* Δ*galU* was grown in HTMC at 37°C in a 5 L fermenter to high densities of OD 30 (B1) and OD 39 (B2) and GMMA were purified using 2-step TFF. To determine the amount of GMMA in the retentate of the 0.1 µm TFF (purified GMMA) GMMA were separated from soluble proteins by ultracentrifugation. After ultracentrifugation, the pellets (GMMA) were resuspended in the initial volume of the centrifuged material to normalize all samples to fermentation volume. Equivalent volumes of the retentate before ultracentrifugation (1), the resuspended GMMA pellet (2), and the supernatant of the ultracentrifugation (3) were separated by SDS-PAGE (12% PA). The retentates were found to contain almost exclusively GMMA (2) as determined by the strong reduction of soluble proteins (3). In addition, the protein pattern in GMMA from the 2 fermentations was very similar suggesting good reproducibility of the process. Minor differences in the visible amount of proteins are highlighted by arrows.
(TIF)
