# Supplementary figures and images for: High Yield Production Process for Shigella Outer Membrane Particles
Source: PLoS One. 2012 Jun 6;7(6):e35616. doi: 10.1371/journal.pone.0035616 (PMC3368891; doi:10.1371/journal.pone.0035616)

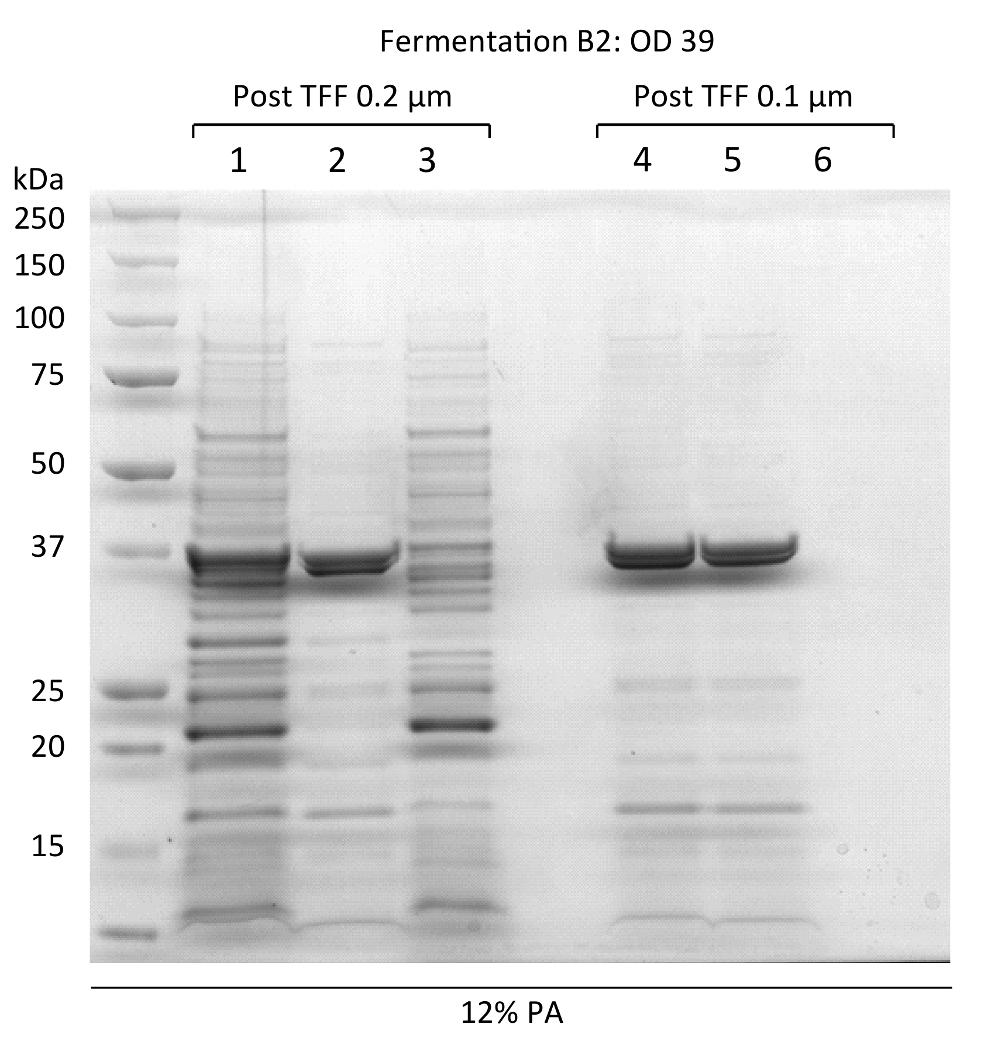

Supplement: Figure S1 — GMMA enrichment and purity after TFF without diafiltration of the biomass. GMMA were purified from a 5 L fermentation culture of S. sonnei ΔtolR ΔgalU grown in HTMC at 37°C to OD 39 (fermentation B2 in Table 3) using 2-step TFF. In the first step, the culture supernatant which contains the GMMA was separated from the bacteria using a 0.2 µm filter without further diafiltration of the biomass. To determine the amount of GMMA in the permeate GMMA were separated from soluble proteins by ultracentrifugation. After ultracentrifugation, the pellet (GMMA) was resuspended in the initial volume of the centrifuged material to normalize all samples to fermentation volume. Equivalent volumes of the 0.2 µm filtrate before ultracentrifugation (1), the resuspended GMMA pellet (2), and the supernatant of the ultracentrifugation (3) were separated by SDS-PAGE (12% PA) and showed a large amount of soluble proteins (3) in comparison to GMMA-associated proteins (2) to be present in the post 0.2 µm TFF permeate. In the second TFF step, GMMA were separated from soluble proteins using a 0.1 µm filter. The retentate (4) was analyzed by ultracentrifugation as described above and was found to contain almost exclusively GMMA (5) as determined by the strong reduction of soluble proteins (6). The high recovery rate of 83% in this process (see Table 3) is reflected in the similar strength of the visible protein bands in lane 2 (GMMA in the 0.2 µm TFF filtrate) and lane 5 (GMMA in the 0.1 µm retentate). (TIF) [file pone.0035616.s001.tif]

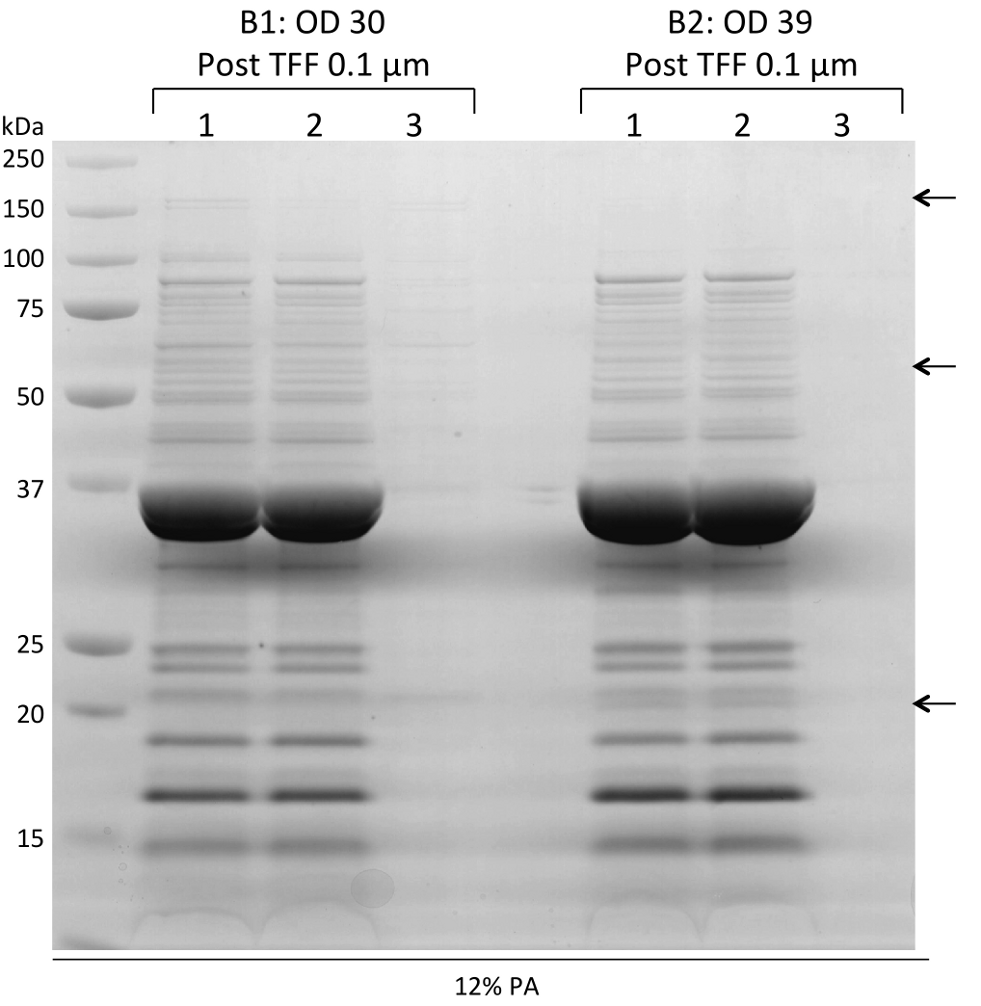

Supplement: Figure S2 — Reproducibility of purity and protein composition of GMMA obtained by the high yield production process. S. sonnei ΔtolR ΔgalU was grown in HTMC at 37°C in a 5 L fermenter to high densities of OD 30 (B1) and OD 39 (B2) and GMMA were purified using 2-step TFF. To determine the amount of GMMA in the retentate of the 0.1 µm TFF (purified GMMA) GMMA were separated from soluble proteins by ultracentrifugation. After ultracentrifugation, the pellets (GMMA) were resuspended in the initial volume of the centrifuged material to normalize all samples to fermentation volume. Equivalent volumes of the retentate before ultracentrifugation (1), the resuspended GMMA pellet (2), and the supernatant of the ultracentrifugation (3) were separated by SDS-PAGE (12% PA). The retentates were found to contain almost exclusively GMMA (2) as determined by the strong reduction of soluble proteins (3). In addition, the protein pattern in GMMA from the 2 fermentations was very similar suggesting good reproducibility of the process. Minor differences in the visible amount of proteins are highlighted by arrows. (TIF) [file pone.0035616.s002.tif]
